# Transcriptional Regulation of Aerobic Metabolism in *Pichia pastoris* Fermentation

**DOI:** 10.1371/journal.pone.0161502

**Published:** 2016-08-18

**Authors:** Biao Zhang, Baizhi Li, Dai Chen, Jie Zong, Fei Sun, Huixin Qu, Chongyang Liang

**Affiliations:** 1 Institute of Frontier Medical Science of Jilin University, Changchun 130021, P.R. China; 2 NovelBio Bio-Pharm Technology Co., Ltd, Shanghai 200000, P.R. China; National Renewable Energy Laboratory, UNITED STATES

## Abstract

In this study, we investigated the classical fermentation process in *Pichia pastoris* based on transcriptomics. We utilized methanol in pichia yeast cell as the focus of our study, based on two key steps: limiting carbon source replacement (from glycerol to methonal) and fermentative production of exogenous proteins. In the former, the core differential genes in co-expression net point to initiation of aerobic metabolism and generation of peroxisome. The transmission electron microscope (TEM) results showed that yeast gradually adapted methanol induction to increased cell volume, and decreased density, via large number of peroxisomes. In the fermentative production of exogenous proteins, the Gene Ontology (GO) mapping results show that *PAS_chr2-1_0582* played a vital role in regulating aerobic metabolic drift. In order to confirm the above results, we disrupted *PAS_chr2-1_0582* by homologous recombination. Alcohol consumption was equivalent to one fifth of the normal control, and fewer peroxisomes were observed in Δ0582 strain following methanol induction. In this study we determined the important core genes and GO terms regulating aerobic metabolic drift in *Pichia*, as well as developing new perspectives for the continued development within this field.

## Introduction

Many yeast genome studies revealed the various physiological processes. *Saccharomyces cerevisiae* has been used as a general model to explain the stress response in cells at the transcriptional level under some certain conditions, such as high temperature, anoxia and nitrogen source starvation [1–9].

However, fermentation studies involving transcriptomics of engineered yeast, such as *Saccharomyces cerevisiae*, *Pichia pastoris* and *Hansenula* used in drug production, are rare; although these strains have been used in the production for nearly 30 years. The effect of fermentation parameters on yeast occurs over a time period. Key changes in transcriptomics of yeast cell occur during the fermentation, which is crucial for clarification of the role of core genes in the entire process. It provides a theoretical basis for further optimization of fermentation at the genome level. Kristin Baumann et al. [10] modified the genome of *Saccharomyces cerevisiae* for BMS1 over-expression and knocked out some of the genes to improve the efficiency of ribosome biosynthesis, to increase the yield of recombinant target proteins.

The soluble expression of more than 100 exogenous proteins has been achieved in *P*. *pastoris*. *Pichia* yeast has been used in fermentation for more than 30 years, only minor modifications have been made with various optimization methods recent years [11]. Data for significant process improvement are not available yet, and no related theories have been reported. Currently, Sauer and his co-workers [12] have compared the role of hypoxic pressure stress on *Pichia* yeast transcriptomics. However, these studies did not include the transcriptomics of *Pichia* yeast in fermentation.

Fermentation is an intermittent and steady biological process. In this process, yeast migrate from one steady state to another steady state. This migration involves changes at the level of transcriptomics, proteomics and metabolomics. Therefore, optional optimizing ways can be investigated by these changes at different levels during fermentation and the key migration factors such as the core genes and small molecular substrates.

Here, we designed an independent microarray to investigate the transcriptomics related to key stages in the classical fermentation process of *P*. *pastoris*. We provide aditional findings and detailed evidence for the transcriptome of *Pichia* methanol adaptation focusing on redox mechanisms in metabolism. The ultrastructure of yeast provides us with a better explanation underlying the dramatic changes in cell structure during fermentation.

## Materials and Methods

### Strains and strain engineering

The *P*. *pastoris* GS115 pPICZaArHSA strain was used as the starting strain. Recombinant human serum albumin (rHSA) cDNA was amplified by PCR from human liver cDNA library. The PCR product was digested with EcoRI and NotI to obtain the rHSA cDNA fragment and then ligated into linearized vector pPIC9k. The ligated plasmid pPIC9 k/rHSA was transformed into competent *E*. *coli* JM109 strain, and selected on Luria-Bertani (LB) agar plates (1% tryptone, 0.5% yeast extract, 1% NaCl, w/v, pH 7.0) containing 100 μg/mL ampicillin. The positive transformants harboring expression plasmid pPIC9 k/rHSA were selected and the sequence of the isolated plasmid was verified by EcoRI/NotI digestion and sequencing. The plasmid pPIC9K/rHSA was digested with SalI. The linear plasmid DNA product was transformed into the *P*. *pastoris*GS115 by pulsed electroporation at 1.5 kV, 25 lF and 200 Ω. A detailed description of transformation and selection of recombinant *P*. *pastoris* with high expression capacity of fusion protein is available [13].

### Fermentation process

The large-scale expression was conducted in a 40 L Bioflo-510 fermentor (NBS Co., USA). The process of fermentation referred to Chester’s report [11], it was described in Table 1. it was described in Table 1. The preparation method of the methanol solution is described as below:

50% glycerol containing 12 ml PTM1 Trace Salts per liter of glycerol.

100% methanol containing 12 ml PTM1 Trace Salts per liter of methanol.

**Table 1:** Key events in fermentation.

Time-Point	Description
1 (12h)	Logarithmic growth phase
24h	Depletion of glycerol in the fermentation medium, initiation of glycerol-fed biomass generation.
2 (30h)	End of glycerol-fed phase, and starvation of carbon for 40 min. Initiation of methanol-fed phase.
3 (48h)	End of methanol-fed phase, cells growing on methanol as the sole carbon source.
4 (90h)	Peak of rHSA expression rate.
5 (108h)	Decrease of rHSA expression to 220 mg /L, and end of fermentation.

The consumption of methanol was measured by Sartorius customized measure system.

### Genomic DNA and total RNA preparation

The *P*. *pastoris* GS115 microarray encompasses well-known and predictable *P*. *pastoris* GS115 genes and transcripts. Coupled with NCBI gene prediction processes and Agilent's probe selection, the design delivers increased data quality as well as less redundant gene coverage. The 5040 *P*. *pastoris* GS115 genes and transcripts were annotated. Each gene has one probe and most probes have 3 replications. The sequence content was sourced from NCBI BioProject PRJNA39439. All the representative probes were designed by Agilent's eArray. The sequence orientation, accuracy, and clustering assembly classification was validated with *P pastoris* GS115.

Cells were subjected to further extractions, and 9 mL of culture were mixed with 5 mL of freshly prepared chilled 5% (v/v) phenol (Sigma) solution in absolute ethanol, centrifuged at 4°C and 12, 000 rpm for 5 min. The harvested cells were stored at -80°C until extraction.

The RNA extractions were performed with RNeasy Mini Kit (Qiagen) following the manufacturer’s protocol of enzymatic extraction using lyticase (Sigma). RNA samples were quantified and analysed for purity using Experion RNA StdSens Analysis Kit (Bio-Rad) with a RQI between 8.8 and 9.9. The GenomeOligo microarray of *P pastoris* was custome designed by Agilent corporation, and the detailed description is provided in S1 File.

### TEM and gene disruption

Yeast cell samples were fixed with 2.5% glutaraldehyde at 4°C for 12 h, then rinsed three times with phosphate buffer and post-fixed with 1% osmium tetroxide at 4°C for 2 h. Thereafter, the samples were serially dehydrated in ethanol, and embedded in Epon812. Thin sections of the samples were obtained on 200-mesh copper grids and stained with uranyl acetate and lead citrate. Samples were observed with a JEM-2100 transmission electron microscope [14].

### Gene disruption

Transformation of *P*. *pastoris* GS115 strain with linearized PAS_chr2-1_0582 fragment obtained by ScaI digestion was carried out in Gene Plus. PAS-chr2-1_0582 disruptant transformants were obtained using intergenic primers [15] by GENSCRIPT Co., Ltd.

### Microarray hybridization and data analysis

Total RNA was submitted to NovelBio Bio-Pharm Technology Co., Ltd for sample processing and chip hybridization according to the manufacturer’s instructions. The arrays were scanned with the Agilent Microarray Scanner (Agilent p/n G2565BA), the data was extracted with Agilent Feature Extraction software. Normalization was carried out by the robust multi-array average (RMA) method [16,17]. Differential expression genes were determined from statistical outcomes by testing for association with biological process gene ontology (GO) terms with a web-based software GOEAST [18]. Fisher’s exact test was used to classify the GO category, and the false discovery rate (FDR) was calculated to correct the P value. The network was produced introducing the co-expression in *Cytoscape* (a bioinformatics software platform for visualizing molecular interaction networks) [19].

### qRT-PCR assay

Quantitative real-time PCR was carried out in 20 μL reactions using semi-skirted iQ 96-well PCR plates and iQTMSYBR® Green supermix (Bio-Rad). Samples were measured in triplicates and the standards were measured in duplicates on the iCycler Thermal Cycler (Bio-Rad). A non-template control was run in every experiment for each of the primer pairs to avoid detection of unspecific priming. The reactions were incubated at 95°C for 10 min to activate Taq polymerase, and were then subjected to a three-step cycling protocol including melting (95°C, 15 sec), annealing (58°C, 15 sec) and extension (72°C, 30 sec) for a total of 40 cycles. Each extension was followed by data collection at 72°C. After a final extension of 5 min at 72°C, a melt-curve profile was generated by data collection during 81 cycles starting at 55°C to 95°C, with 0.5°C increments/cycle (1-sec intervals).

## Results

### Fermentation of rHSA production induced by methanol in *P pastoris*

The traditional methanol-induced *Pichia* yeast fermentation, comprises five extremlely important phases witihin the cell. These were the essential nodes to determine the status of the strain, as well as the production of recombinant proteins.

The five nodes correspond to the logarithmic growth phase in the chemostat cultivation, limiting carbon source replacement, the initial stage of yeast adaption to methanol, the high-peak production stage of recombinant proteins, and cell senescence and product degradation. Therefore, the yeast cells at these five time-points were selected for genomic study.

**Fig 1:**
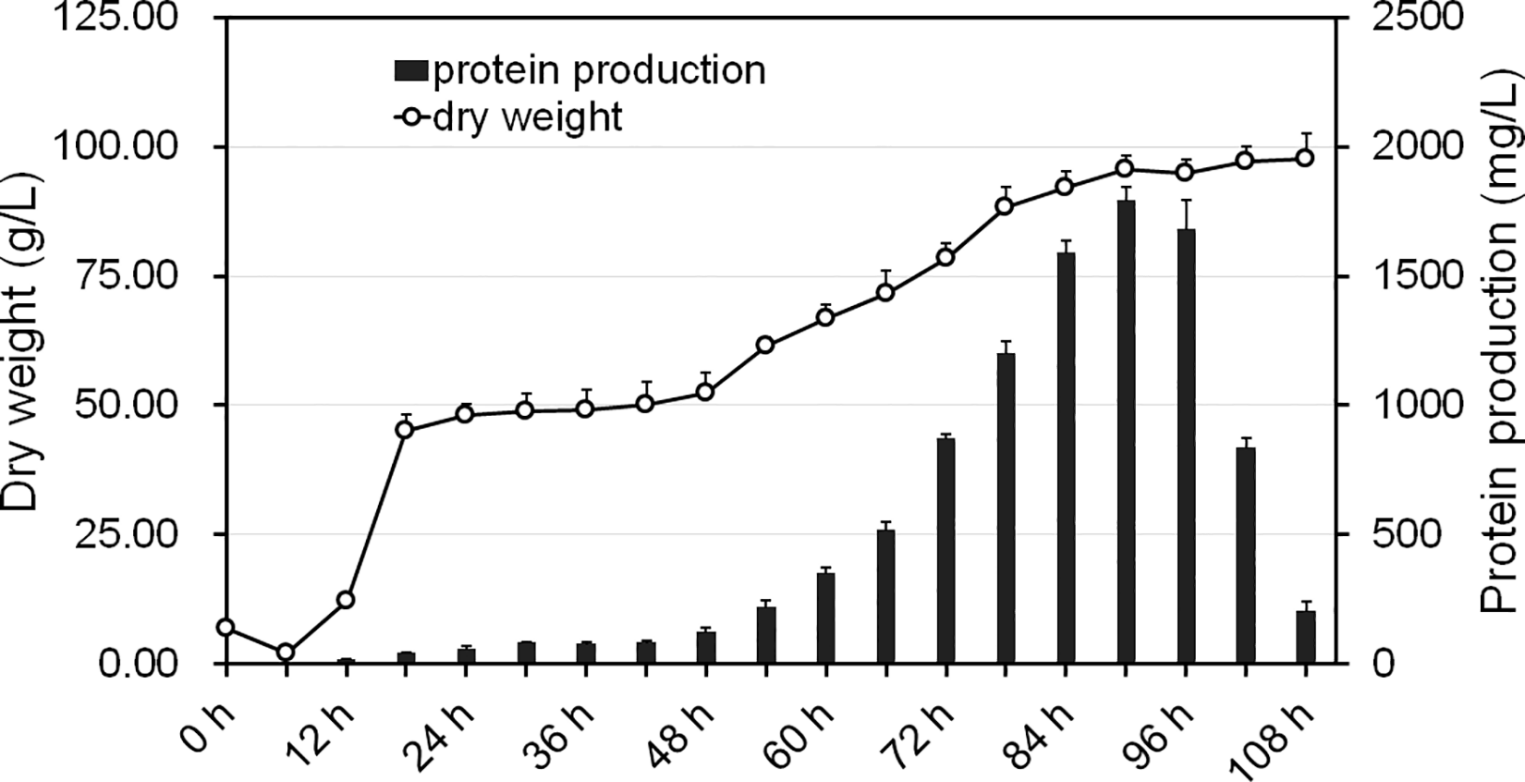
Fermentation of rHSA expression induced by methanol in *P*. *pastoris*. The spot line represents changes in cell dry weight, and the columns indicate the rHSA levels. The whole fermentation cycle lasted for 108 h. The cell growth stage (G stage G0h-G24h) lasted 6h-30h, the glycerol fed-batch stage (GB stage GB0h-GB6h) lasted 30h-36h, and the methanol fed-batch stage 36h-108h (MB stage MB0h-MB72h).

*P*. *pastoris* with recombinat rHSA expression was induced by methanol and was considered the model for whole fermentation cycle, which lasted for 108 hours. The process consisted of three main components: cell growth stage (G stage, lasting for 24 h), glycerol fed-batch stage (GB stage, lasting for 6 h) and methanol fed-batch stage (MB stage, lasting for 72 h). As shown in Fig 1, rHSA expression appeared in the G stage and GB stage, although the amount was low, at less than 100 mg/L. The maximum peak of rHSA expression appeared at the 54^th^ h in the MB stage (MB 54h). The rHSA concentration in the tank at the highest expression was 1800mg/L. However, it started to decrease and down to only 220 mg/L at the 108^th^ h (MB 72h). The logarithmic growth phase appeared in the 12^th^ hour (G6h) with the carbon source replaced by methanol at the 30^th^ h (G24h). In order to ensure glycerol depletion in the fermentation broth, the yeast was subjected to carbon deprivation for 40 min. The expression level increased tremendously at the 48^th^ h (MB12h), indicating yeast adaptation to the methanol. Based on the the protein expression level, the highest peak of rHSA expression rate occurred between the 84^th^ hour and 90^th^ h (MB48h to MB54h). The yeast cells at 5 time points, namely, G6h, G24h, MB12h, MB54h, MB72h, were chosen for investigation in the traditional methanol-induced *Pichia* yeast fermentation.

### Transcriptomics of *Pichia* yeast fermentation: overview

We designed 5040 transcript probes and customized the microarray to Agilent S1 File. The five time points during the fermentation were detected using whole-genome expression microarray. Briefly, three replicates were studied on yeast cell sample of each time point. The Quantile method was used for the standardization, and the microarray data were uploaded to the GEO (GSE56873).

**Fig 2:**
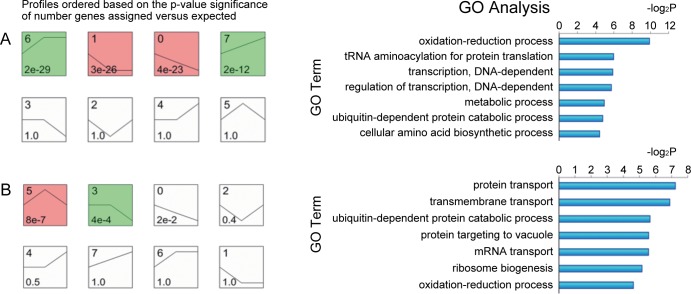
Main GO terms affected by differential genes and patterns during the transition from time point 1 to 3 and 3 to 5. Each box represents one pattern of a model expression profile. The upper number in the profile box is the model profile number, and the lower one is the p-value used to summarize the different gene expression patterns. (A) The 1–3 time points in the 8 expression patterns were clustered, respectively. Genes expressed during the 1–3 time points were distributed in No. 6,1, 0 and 7 pattern (p<0.05). The core gene during the first three time points was associated with redox function. (B) The 3–5 time points in the 8 constructed expression patterns were clustered, respectively. The main GOTerms were affected by differential genes. Genes expressed during the 3–5 time points were distributed in the No.3 and No.5 pattern (p<0.05). The core gene during the 3–5 time points was associated with protein transport.

The expression patterns of differentially expressed genes during the 1–3 time points and 3–5 time points in the 8 constructed expression patterns were clustered, respectively. Fisher’s exact test showed that differentially expressed genes during the 1–3 time points were significantly distributed in No. 6, 1, 0 and 7 pattern. There were 125 differentially expressed genes in No.0, 228 in No.1, 141 in No. 6 and 64 in No. 7 (Fig 2A), whereas differentially expressed genes during the 3rd to 5th time points were distributed in No.5 and No.3 pattern. All the differentially expressed genes were predominantly in No.3 and No.5 pattern (color box) (P < 0.05)(Fig 2B)

Significant functional analysis was provided for the analysis of 833 differentially expressed genes during the 1–3 time points and 3–5 time points (P < 0.05) (Fig 2A, Fig 2B). The results suggest that the redox processes were affected significantly. These characteristics represented functional differentiation and the differentially expressed genes related to redox processes formed a GO-Genes-Tree. The dynamic co-expression network of 192 genes affecting significant function during the 1–3 time points and 231 genes significantly affecting the functions during the 3–5 time points were constructed. The gene co-expression relationship was determined by calculating the Pearson correlation coefficient among the genes. The connected nodes of each gene (Degree) were calculated based on graph theory, as well as the core (K-Core) of the network containing the gene. The results showed that the maximum value of K-Core during the 1^st^ to 3^rd^ time points was 11. The genes in the network core included *PAS_chr1-4_0165*, *PAS_chr2-2_0041*, *PAS_chr2-2_0145*, *PAS_chr3_0317*, *PAS_chr4_0243* and *PAS_chr1-3_0244* (Table 2). The maximum value of K-Core during the 3–5 time points was 5 and the genes in the network core included *PAS_chr1-1_0354*, *PAS_chr4_0561*, *PAS_chr4_0836*, and *PAS_chr1-3_0016* (Table 2). The foregoing results suggest that the network density was higher and a potent synergetic gene co-expression relationship existed among the genes during the 1–3 time points. The network density declined greatly and the synergistic gene co-expression among the genes was very low during the 3–5 time points. Redox processes (GO:0055114) were the main functions associated with by the core gene during the 1–3 time points. The main functions associated with the core gene during the 3–5 time points included protein transport (GO:0015031), indicating that the redox function was the predominant feature during the initial 3 time periods (Fig 2A), and protein transport was the predominant feature during the 3–5 time points (Fig 2B).

**Table 2:** Core genes of co-expression network in two time periods.

GeneSymbol	Description	Degree	K-Core
	1^st^ to 3^rd^ time points		
PAS_chr1-4_0165	Class E protein of the vacuolar protein-sorting (Vps) pathway	15	11
PAS_chr2-2_0041	Serine/threonine protein kinase, subunit of the transcription factor TFIIH	15	11
PAS_chr2-2_0145	Isozyme of methylenetetrahydrofolate reductase	15	11
PAS_chr3_0317	Farnesyl-diphosphate farnesyl transferase (squalene synthase)	15	11
PAS_chr4_0243	Protein of unknown function	15	11
PAS_chr1-3_0244	Cytoplasmic and mitochondrial histidine tRNA synthetase	14	11
	3^rd^ to 5^th^ time points		
PAS_chr1-1_0354	Allantoin permease	9	5
PAS_chr4_0561	Mitochondrial intermembrane space protein, forms a complex with TIm8p	8	5
PAS_chr4_0836	Polyamine transport protein, recognizes spermine, putrescine, and spermidine	8	5
PAS_chr1-3_0016	Multifunctional enzyme of the peroxisomal fatty acid beta-oxidation pathway	7	5

### Core genes in carbon source replacement

**Fig 3:**
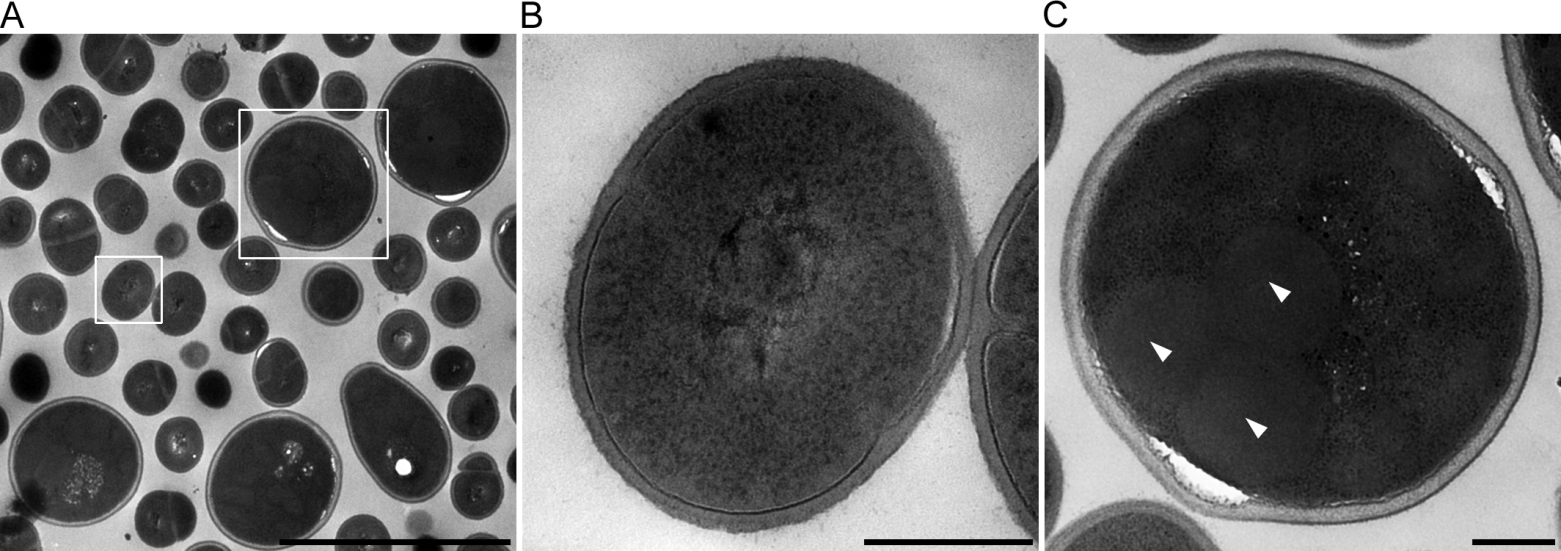
Cellular changes during carbon source replacement. A: Cells during carbon source replacement. B: Common cell. C: Methanol treatment increased cell volume and peroxisome production.

The cell volume increased several-fold and the density decreased. A large number of peroxisomes appeared during yeast fermentation of methanol (Fig 3). The changes entailed comprehensive analysis of the transcriptome microarray data points and important GO terms, pathways and core genes.

We provide additional findings and detailed evidence supporting the transcriptome profile of *Pichia* methanol adaptation. The two processes focus on related redox metabolism. Ultrastructural imaging of yeast elucidates the dramatic changes underlying cell structure in fermentation.The genes associated with the four expression patterns indicated in the previous section were analyzed by computing the co-expression network and functional analysis. The results showed that the main functions were consistent with previous analysis, indicating that the gene distribution pattern within the co-expression network selected was consistent with the overall differential gene expression.

**Fig 4:**
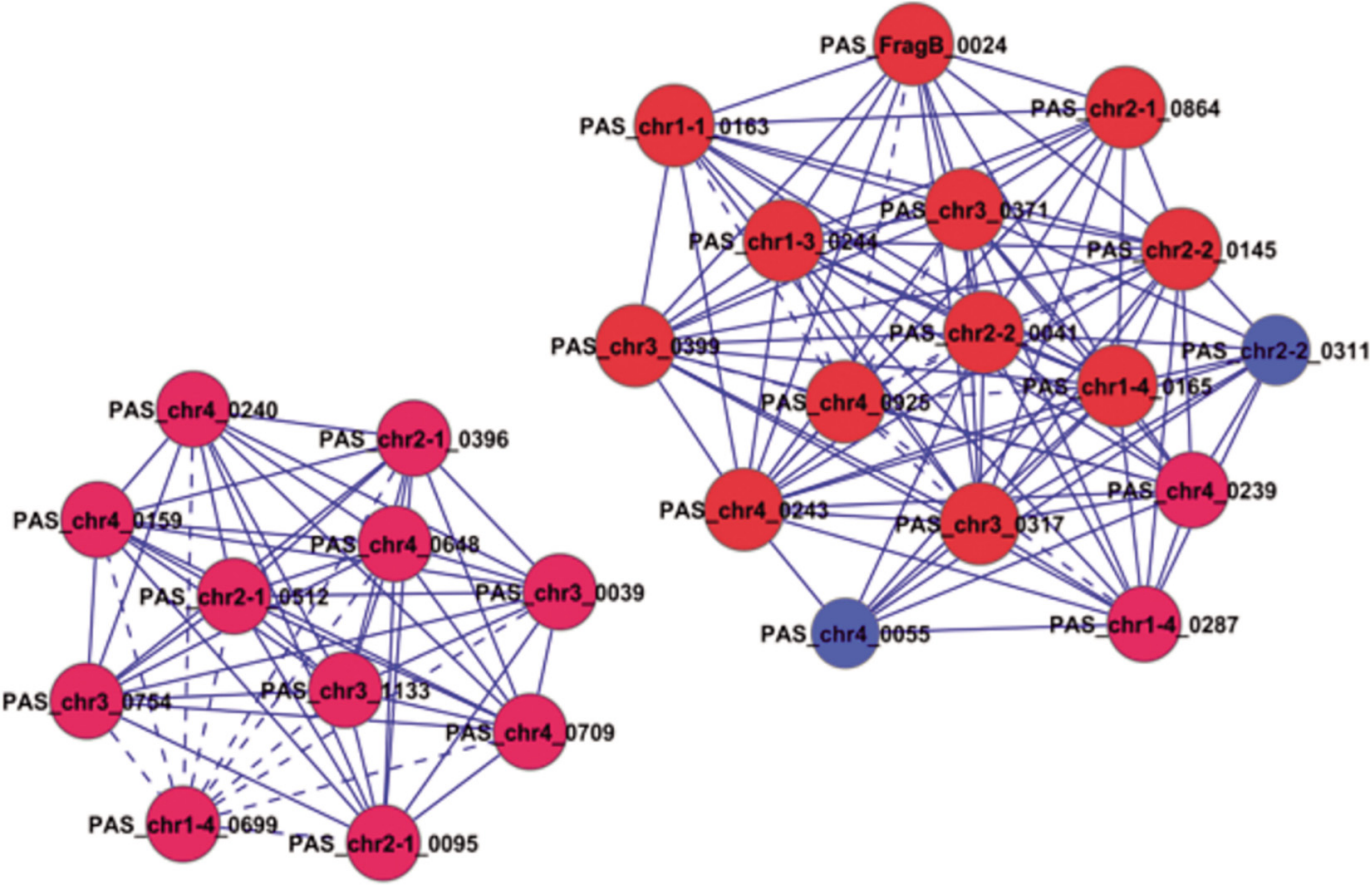
Core genes in differential gene co-expression network during the transition from time point 1 to 3. Cycle node represents gene, the real line and dotted line between two nodes represent direct and indirect interactions between genes respectively. The red nodes represent genes with the K-core value greater than or equal to 10, the blue nodes represent genes with the K-core value less than 10.

Co-expression analysis underscores the significance of a gene with the k-core value in the network to determine the role of the gene in the process from time point 1 to 3. The co-expression network center during the 1-3-stage comprised 29 genes with k-core values greater than or equal to 10. The 29 genes were distributed in a single sub-network (Fig 4) in the No. 0 and No.1 expression pattern, with both expression patterns showing a declining expression pattern. The functions were related to transcriptional regulation indicating that during the process of carbon source replacement, carbon source starvation at time point 2 (G24h) significantly affected the regulatory function in cell growth, which was not restored until time point 3 (MB12h). The finding was consistent with the cell growth curve during the fermentation process displayed in Fig 1, suggesting that the anabolism of *P*.*pastoris* was less sensitive to methanol than glycerol. Fig 4 suggests that *PAS_chr1-4_0165*, *PAS_chr2-2_0041*, *PAS_chr2-2_0145*, *PAS_chr3_0317*, *PAS_chr4_0243* and *PAS_chr1-3_0244* contributed to core regulation of co-expression network.

Additionally the No.6 and No.7 patterns manifesting an elevated expression represent primarily the expression of 9 genes involved in ascospore formation, which were regulated upward. This function occurred downstream during the cell growth, and the up-regulated expression occurred as long as the yeast was in the growth stage. Other comparatively significant upregulation of differential genes was associated with translation, transmembrane and transport dipeptide transport, which were related to protein expression. The results were consistent with the rHSA secretion expression curve in the experiment. The data suggest that although the protein synthesis in the microbial cells in the logarithmic growth phase (time point 1, G6h) was very high, it was still lower than in the inhibitory phase of yeast. AOX1 induced by methanol triggered protein synthesis to drive early expression.

### Role of transcriptomics in decreased rHSA expression during methanol-induced phase

Pattern No. 5 showed elevated gene expression, followed by a decline, which was consistent with altered cell growth rate and rHSA expression. Both patterns of expression attained the highest value at time point 4 (MB 54h). The highest peak of rHSA expression rate was at MB54h, followed by decreased methanol utilization and decline in rHSA yield. Cytoplasmic translation, protein folding in the endoplasmic reticulum, methylation, protein targeting to endoplasmic reticulum (ER) and signal peptide processing GO terms were found in the functional analysis of genes in the 5th pattern. The efficiencies of protein translation, and protein folding in the endoplasmic reticulum and protein targeting declined, as well as the efficiency of intracellular signal peptide process following protein folding. These decreases in function might be an important factor underlying the decreased methanol-induced protein expression levels. The differences in GO terms involved nucleic acid methylaion. The data analysis showed that methylation occurred in 10 genes among the 58 genes involved. The methylation of *TRM9* and other genes declined at time point 4, suggesting cellular aging during the late stage of fermentation [20–24].

**Fig 5:**
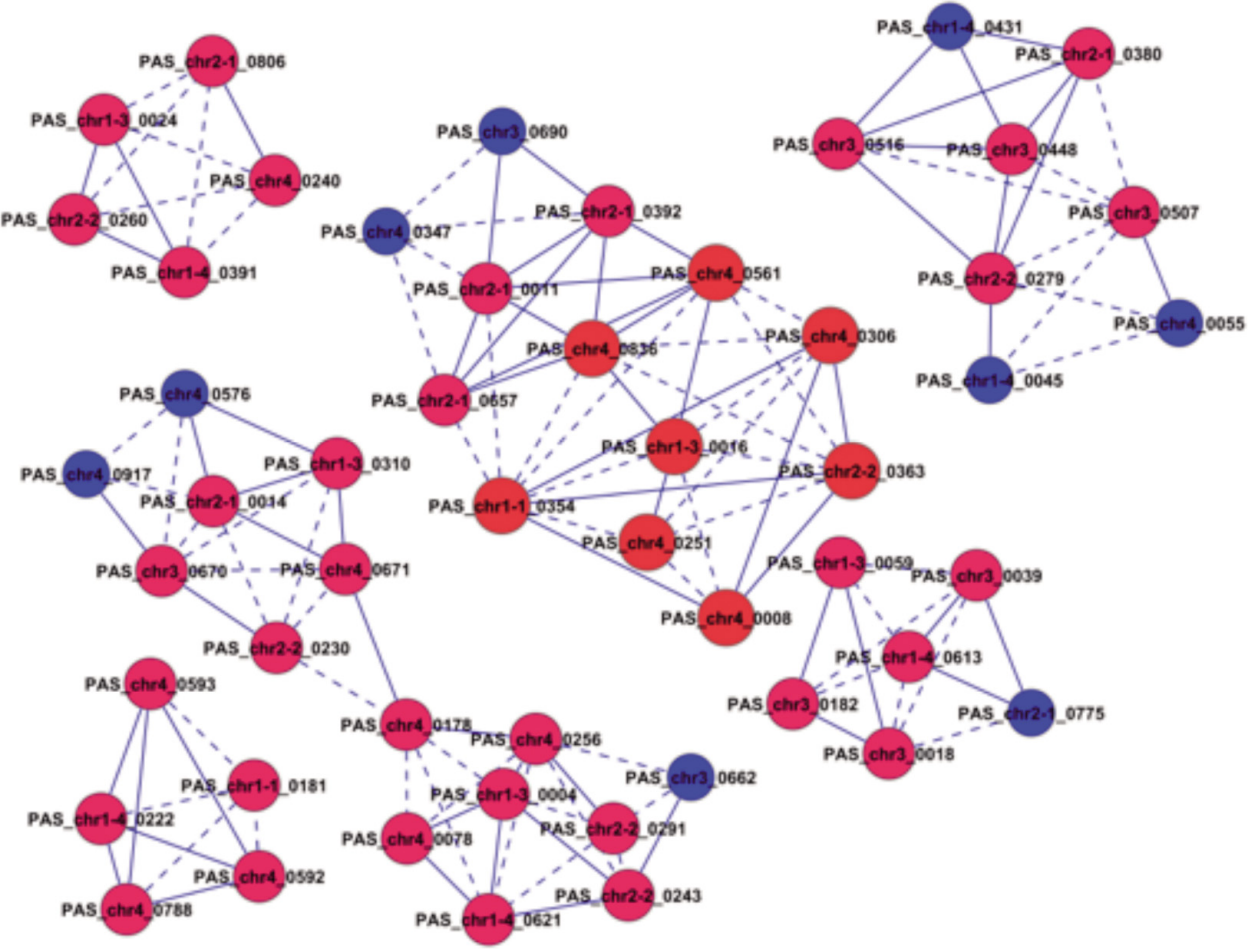
Co-expression network core of differential genes. Time Point 3 to 5. Cycle nodes represent genes; the real line and dotted line between two nodes represent direct and indirect interactions between genes, respectively. The red nodes represent genes with the K-core value greater than or equal to 4, the blue nodes represent genes with the K-core value less than 4.

The distribution pathways included RNA transport and protein export, associated with protein expression and transport. The process from time points 3 to 5 basically represented full genome data in the whole expression process induced by methanol. Most of differentially expressed genes can be classified into two ancestral functions, protein transport and transmembrane transport. The center of the co-expression network of all the differential genes is shown in Fig 5: the core genes were *PAS_chr1 1_0354* (No. 3 pattern, reverse regulation), *PAS_chr4_0561* (No.5 pattern) and *PAS_chr4_0836* (No.5 pattern). Transmenbrane transport was the key function in in recombinant protein expression.

### Decline in aerobic metabolism in the late stage of methanol-induced fermentation

**Fig 6:**
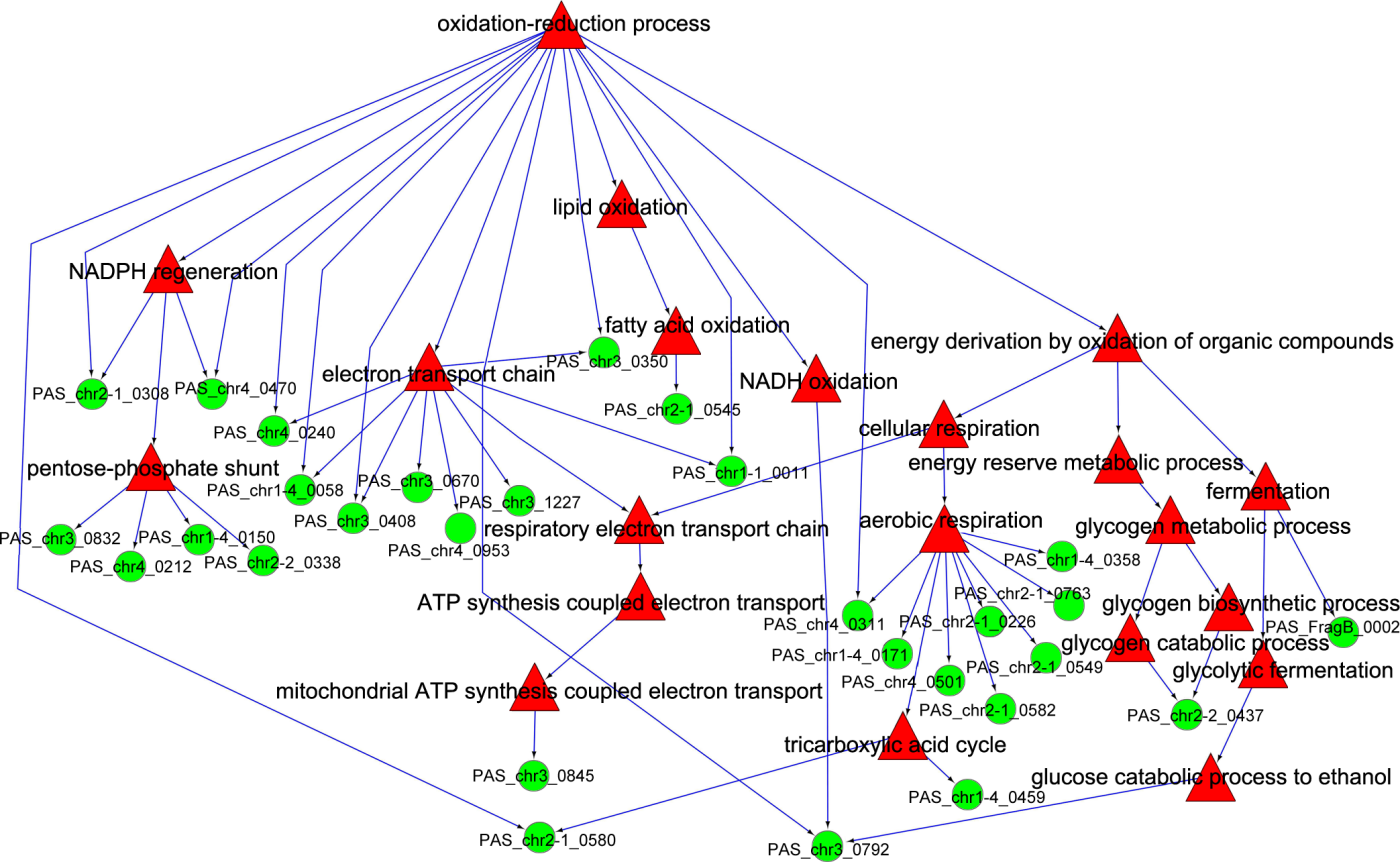
Distribution of differential genes in the redox function tree during the transition from time point 3 to 5. The green cycle node represents gene, and the red triangle nodes represent GO term.

Although the exhaust gas analysis results showed that oxygen uptake rate (OUR) detected the extreme value during the transition from time point 3 to 5, we suspected that the intensity of aerobic metabolism also changed in the process from time point 3 to 5. We also suspected that the range of this change might be outside the valid range of detection in the exhaust gas analysis. The mapping results of the redox function tree and the differential genes are shown in Fig 6. The differences in the distribution of different genes in the function tree were not substantial. However, the expression patterns of *PAS_chr2-1_0226*, *PAS_chr4_0501*, *PAS_chr2-1_0549*, *PAS_chr2-1_0763* and *PAS_chr2-1_0582* in the GO term of aerobic respiratory were contrary to those in the previous stage suggesting that the level of aerobic metabolism might decline partially during the late stage of methanol-induced fermentation.

### *PAS_chr2-1_0582* is the core gene regulating aerobic metabolism drift and ultrastructural changes in yeast

Redox mechanismsin the *Pichia* yeast fermentation are an interesting aspect of investigation. Guo Meijin *et al* reported the linear drift from glycerol metabolism to anaerobic and aerobic metabolism in *P*. *pastoris* chemostat culture stage involving the linear metabolic migration from gluconeogenesis and pentose phosphate pathway to glycolysis and tricarboxylic acid cycle [25]. The extreme value of aerobic metabolism throughout the methanol restrictive feeding induced phase, was consistent with the analytical results in this study. The analysis of genomic data also showed that the redox function was not the key GO term affected in this stage. Enfors SO *et al* have mapped the whole methanol oxidation process in yeast cells, involving the key role of redox function in the expression of recombinant proteins in *P*. *pastoris* [26].

In view of the important role played by redox mechanisms in whole yeast fermentation process, a redox function tree was established. The differential genes in two stages, namely time point 1 to 3 and time point 3 to 5, were mapped into the redox function tree, to determine the primary sub-functions and their related genes during the two time periods.

**Fig 7:**
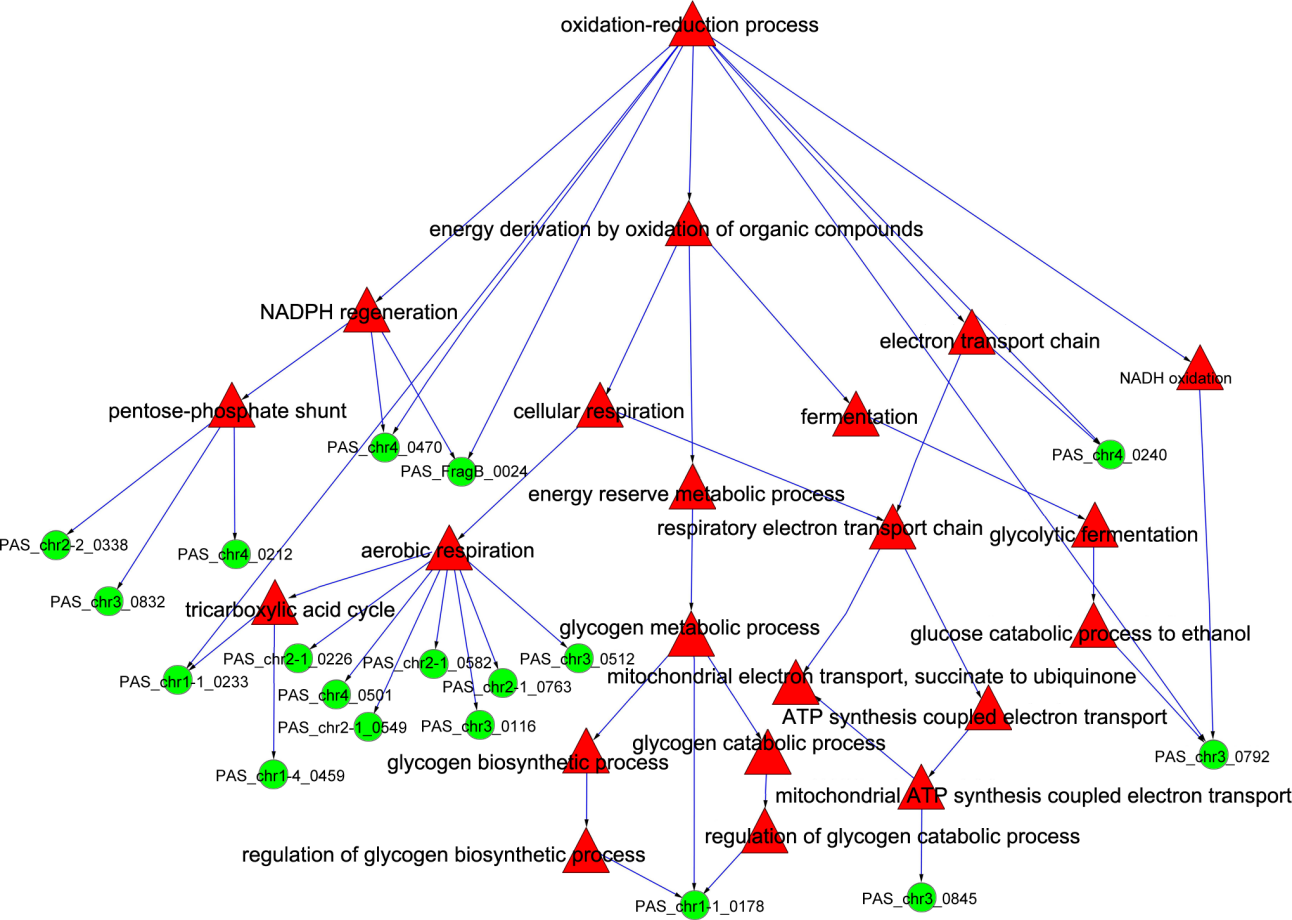
Distribution of differential genes in the redox function tree during the transition from time point 1 to 3. The green cycle nodes represent genes, and the red triangle nodes represent GO term.

Fig 7 suggests that the sub-functions of yeast aerobic respiration and tricarboxylic acid cycle during the period from time point 1 to 3 were maximally affected. The important genes were *PAS_chr2 1_0226*, *PAS_chr4_0501*, *PAS_chr2-1_0549*, *PAS_chr2-1_0763*, *PAS_chr2-1_0582*, *PAS_chr3_0116* and *PAS_chr3_0512*. The combined analytical results depicting the expression analysis and the co-expression network indicated that *PAS_chr2-1_0582* might be the most important gene facilitating the yeast drift from redox function to aerobic respiration.

The gene *PAS_chr2-1_0582* was disrupted by homologous recombination, and the strain was designated as Δ0582. After initial selection of *P*. *pastoris* integrants on minimal dextrose (MD) plates (1.34% yeast nitrogen base without amino acids, 0.00004% biotin, 2% dextrose, 1.5% agar) or YPD (Yeast extract Peptone Dextrose)-Zeocin plates (1% yeast extract, 2% peptone, 2% dextrose, 1.5% agar 50μg/mL Zeocin), successful gene disruption was confirmed by PCR amplification using sequence-specific primers.

**Fig 8:**
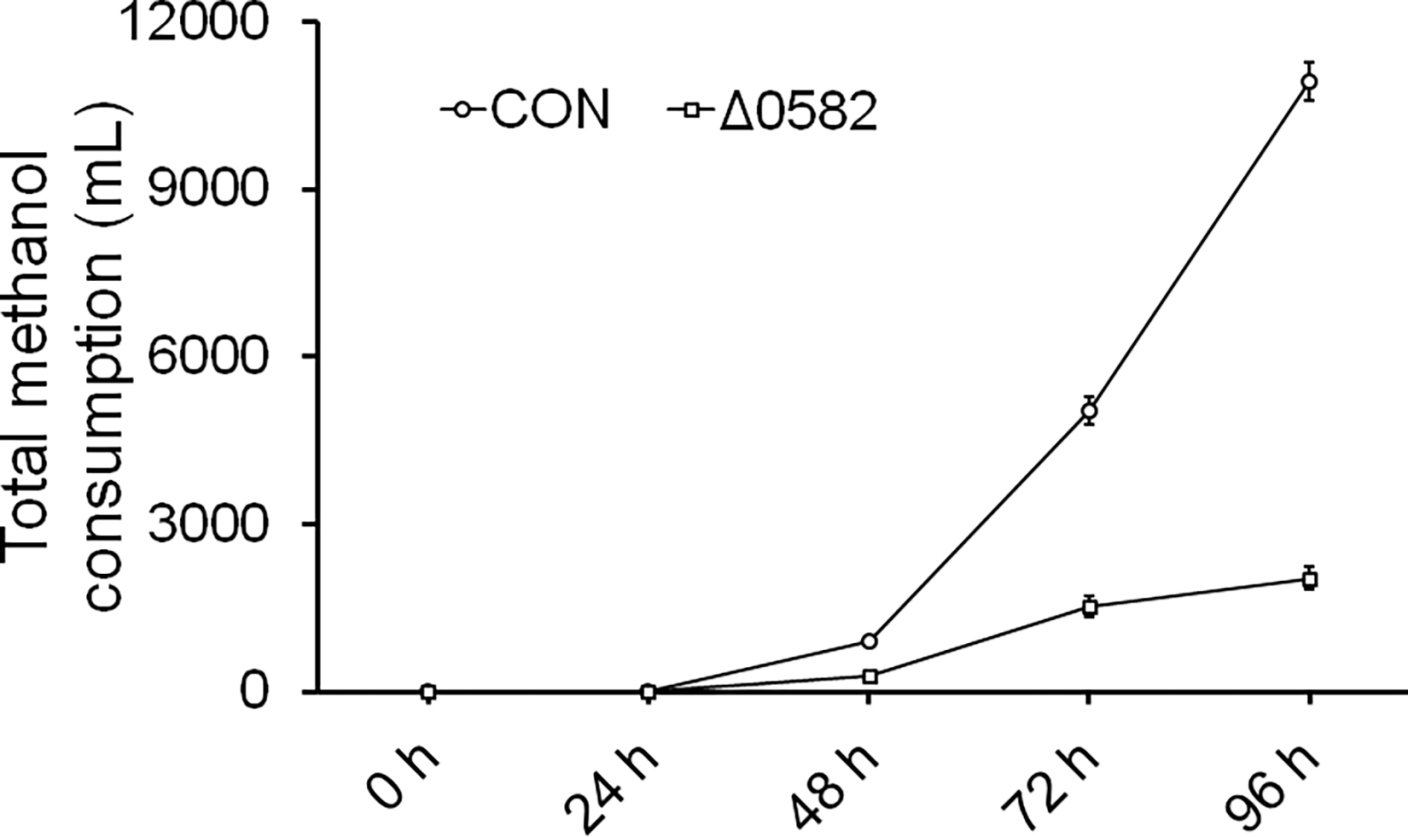
Δ 0582 strains in alcohol fermentation. The methanol consumption of Δ 0582 strains was significantly less than the normal control, equivalent to a fifth of the normal control.

Fig 8 shows that the alcohol consumption in Δ 0582 strains is very low during the fermentation, equivalent to one fifth of the normal control. Microarray data indicated that *PAS_chr2-1_0582* represented the core gene in the co-expression network of redox processes utilizing methanol. According to the GO database, the PAS_chr2-1_0582 represents a Go term (0009060): aerobic respiration, whose functional significance is deduced using BLAST to *Saccharomyces cerevisiae*. Disruption of *PAS_chr2-1_0582* was used to identify the role. The peroxisome represents the primary organelle involved in methanol oxidation in yeast. Compared with the normal control, fewer peroxisomes were observed in the Δ0582 strain that was induced by methanol at 12h using transmission electron microscopy. The results confirm that *PAS_chr2-1_0582* played a vital role in regulating aerobic metabolism.

## Discussion

In the current study, the classical fermentation process was used. Glycerol was replaced by methanol as the restricted carbon source. In the early stages of the study on *P*. *pastoris*, glycerol in the fermentation broth affected the intensity of AOX1 induction, abrogating the expression of exogenous proteins synthesized using AOX1 gene promoter. In this replacement, exhaustion of glycerol is a key step involving methanol addition following glycerol for 30–40 min. After the starvation stage 10–12 h were needed for the yeast cell to adapt to methanol. The genomic data in this study showed that the carbon source replacement in cell growth, the levels of transcription and translation, protein and nucleic acid synthesis were all strongly inhibited. However, the expression of rHSA was found during the glycerol feeding stage. It suggested that AOX1 might be induced by glycerol, corresponding to the recent findings [27]. The finding suggests that traditional carbon source starvation may be an unnecessary and even harmful step in the process. The process in which the combination of glycerol and methanol was fed followed by methanol feeding alone was a rational approach to prevent extreme growth stress in yeast cells during the fermentation process. In addition, the process was in compliance with the engineering principles of fermentation.

The respiratory quotient (RQ) of cells is an important parameter to characterize the physiological status and metabolic pathways of microorganisms. With the increase in the specific growth rate (μ), the RQ of cell metabolism increases linearly (Fig 6) (linear correlation coefficient r = 0. 97). A profound change in the intrinsic physiological metabolism in cells occurs with the changes in specific growth rate [26,27,28]. With the increase in the specific growth rate (μ), the glycerol metabolic flux of the linear migration from gluconeogenesis and pentose phosphate pathway to glycolysis and tricarboxylic acid cycle pathway increases linearly. When the glycerol is catalyzed by phosphoglycerate kinase into dihydroxyacetone phosphate, it enters the glycolytic and tricarboxylic acid cycle pathway, with the release of three molecules of CO_2_. Glycerol is metabolized via glycolytic pathway and pentose phosphate pathway, when the RQ is less than 0.39. Glycerol is metabolized via the glycolytic pathway and tricarboxylic acid cycle pathway when the RQ is greater than 0.625. The values of RQ measured in the practical cell culture are generally significantly lower than the theoretical values. The extent of variation depends on the number of products generated per unit of yeast cells utilizing the substrates.

The electron transport chain is the most important link connecting the four types of redox metabolism. During the carbon source replacement shown in Fig 6 (i.e. chemostat culture process), the energy metabolism resulting from oxidation represented by the axis of the function tree is the most important progenitor function. During this period, the trunk of the function tree, aerobic respiration, gluconeogenesis and electron transport sub-functions are located downstream. Gluconeogenesis is anaerobic metabolism, and the sub-functions include glycogen biosynthesis, glycogen catabolism, regulation of glycogen biosynthesis and regulation of glycogen catabolism. The results show that the four functions are collectively directed at the only core gene, *PAS_chr1 1_0178* resulting in a continuous decline more than ten-fold in PAS expression. Our results demonstrate that *PAS_chr1 1_0178* played a key role in the metabolic decline of gluconeogenesis in the yeast glycerol culture.

The gluconeogenesissub-functions in cellular respiration in the redox function tree include aerobic respiration and respiratory electron transport chain. The number of differential genes in the sub-function of aerobic respiration and co-expression is very high. The analysis of the expression pattern of the core genes indicates that the aerobic respiration is enhanced during this period, which was consistent with current experimental reports. The analysis of the co-expression suggests that *PAS_chr2-1_0582* may be the most important gene underlying the drift of yeast cells from a redox function to aerobic respiration. The *PAS_chr2-1_0582* and *HAP* family genes in *S*. *cerevisiae* show high homology. Current studies in this field suggest that the HAP transcriptional complex is involved in the regulation of functional protein expression related to electron transport chain and the tricarboxylic acid cycle in mitochondria [29–36] suggesting that *PAS_chr2-1_0582* may be similar in function to that of HAP transcription complex. As well, it is one of the most important transcription factors regulating and controlling aerobic metabolism in *P*. *pastoris*.

The other respiratory electron transport chain with the sub-function facilitated the drift between anaerobic and aerobic metabolism. The expression of *PAS_chr3_0845* was enhanced more than 10 times during the transition from time point 1 to 3. The BLAST results showed that the homologous gene of *PAS_chr3_0845* in *S*. *cerevisiae* was *Taz P1*.The role of the gene in phospholipid metabolism has been extensively analyzed. However, no data supporting its auxiliary role in the drift from anaerobic to aerobic metabolism has been found until now.

In the late methanol-induced stage, the expression pattern of differential genes showed that the efficiency of protein translation in the cytoplasm, and protein folding in the endoplasmic reticulum decreased. The changes in the transcriptome may be due to the role of nitrogen starvation and other external factors, which are considered to be a manifestation of life processes in yeast cells. The changes are two-fold. First, the expression pattern of a large number of genes associated with nucleic acid methylation was elevated, and then declined. The cells were aged, and the overall metabolism declined after time point 4, which affected the efficiency of rHSA expression. Second, as shown in Fig 7 the flux of aerobic metabolism drifted to the pentose phosphate pathway, which was anaerobic metabolism. The expressions levels of *PAS_chr3_0832*, *PAS_chr4_0212* and *PAS_chr2-2_0338* with the sub-function in the pentose-phosphate shunt were strongly elevated at time point 5 indicating that the metabolic flux during the late methanol-induced stage increased gradually at the transcriptional level in pentose phosphate pathway. Methanol oxidation requires a large number of oxygen molecules, suggesting the need for a very high intensity of aerobic metabolism in yeast cells. The declining level of oxidative metabolism might affect the methanol-induced AOX1 expression in the peroxisome, affecting the level of protein expression.

We used an independently-designed DNA microarray to study the fermentation in methanol-induced exogenous recombinant protein expression in *P*. *pastoris*. Transcriptomics demonstrated the key functions and the core genes in the two processes of protein expression including restrictive carbon source replacement and methanol-induced expression. This study may suggest a mechanism for further optimization of the classic fermentation process.

## Supporting Information

S1 File Agilent Whole Genome Oligo Microarrays. (DOC)
