# Supplementary material for: Transcriptional Regulation of Aerobic Metabolism in Pichia pastoris Fermentation
Source: PLoS One. 2016 Aug 18;11(8):e0161502. doi: 10.1371/journal.pone.0161502 (PMC4990298; doi:10.1371/journal.pone.0161502)
Supplement: S1 File — (DOC) [file pone.0161502.s001.doc]

**Agilent Whole Genome Oligo Microarrays (one-color)**

# Step 1 Prepare the RNA Sample

**Kits and Reagents:**

TRIzol® Reagent (Invitrogen life technologies)

Biopulverizer(biospec)

Mini-Bead-Beater-16(biospec)

**Procedure:**

1. Homogenization

*a. Tissues*

1. Cryopulverization of tissue using the Biopulverizer
   - Place the BioPulverizer™ in a shallow container and cool thoroughly with either liquid nitrogen.
   - Place prefrozen tissue in the well of the mortar.
   - Remove the mortar from the shallow container, place it on the lab bench and pulverize the tissue with one or two blows to the pestle with a hammer (models 59012MS).
   - Empty the powdered contents for subsequent homogenization or extraction procedures.


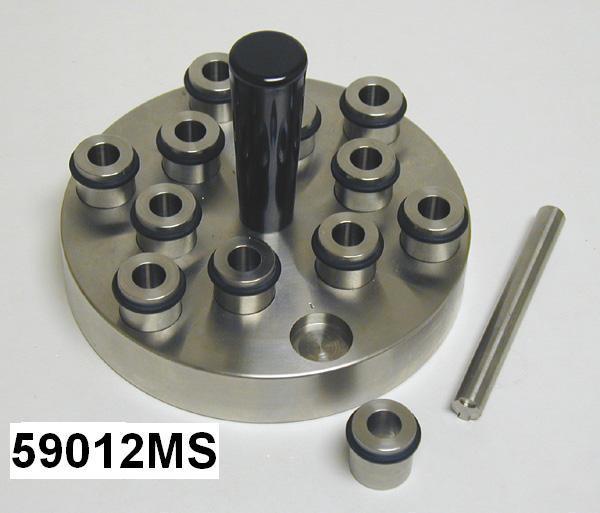


1. Homogenization using the Mini-Bead-Beater-16
   - Fill the 2 ml screw-cap [microtube](http://www.biospec.com/Vials.htm) one-half full with 1.0mm beads. Then add TRIzol and tissue, being sure to fill the microtube almost to the top. Exclude as much air from the microtube as possible. Use screw-cap microtubes with integral o-ring seals in order to eliminate aerosol formation during the homogenization. Be sure there are no beads on the threads of the microtubes when screwing down the cap.
   - Insert 1 to 16 microtubes into the holder. Distribute them symmetrically as you would do with a centrifuge. Screw in the black plastic chamber cap until it is in contact with the tops of the microtube caps. Tighten the lid by hand so the vials are firmly held in place. Do not overtighten.
   - Set the timer depending on the type of sample. Start the machine.

*b. Cells Grown in Monolayer*

Lyse cells directly in a culture dish by adding 1 ml of TRIzol Reagent to a 3.5 cm diameter dish, and passing the cell lysate several times through a pipette. The amount of TRIzol Reagent added is based on the area of the culture dish (1 ml per 10 cm^2^) and not on the number of cells present.

*c. Cells Grown in Suspension*

Pellet cells by centrifugation. Lyse cells in TRIzol Reagent by repetitive pipetting. Use 1 ml of the reagent per 5-10 × 10^6^ of animal, plant or yeast cells, or per 1 × 10^7^ bacterial cells. Washing cells before addition of TRIzol Reagent should be avoided as this increases the possibility of mRNA degradation. Disruption of some yeast and bacterial cells may require the use of a homogenizer.

1. Phase Separation

Incubate the homogenized samples for 5 minutes at 15 to 30°C to permit the complete dissociation of nucleoprotein complexes. Add 0.2 ml of chloroform per 1 ml of TRIzol Reagent. Cap sample tubes securely. Shake tubes vigorously by hand for 15 seconds and incubate them at 15 to 30°C for 2 to 3 minutes. Centrifuge the samples at 12,000 × g for 15 minutes at 4°C. Following centrifugation, the mixture separates into a lower red, phenol-chloroform phase, an interphase, and a colorless upper aqueous phase. RNA remains exclusively in the aqueous phase. The volume of the aqueous phase is about 60% of the volume of TRIzol Reagent used for homogenization.

1. RNA Precipitation

Transfer the aqueous phase to a fresh tube. Precipitate the RNA from the aqueous phase by mixing with isopropyl alcohol. Use 0.5 ml of isopropyl alcohol per 1 ml of TRIzol Reagent used for the initial homogenization. Incubate samples at 15 to 30°C for 10 minutes and centrifuge at 12,000 × g for 10 minutes at 4°C.

1. RNA Wash

Remove the supernatant. Wash the RNA pellet once with 75% ethanol, adding at least 1 ml of 75% ethanol per 1 ml of TRIzol Reagent used for the initial homogenization. Mix the sample by vortexing and centrifuge at 7,500 × g for 5 minutes at 4°C.

1. Redissolving the RNA

At the end of the procedure, air-dry the RNA pellet for 5-10 minutes. Dissolve RNA in 85ul or less RNase-free water by passing the solution a few times through a pipette tip, and incubating for 10 minutes at 55 to 60°C.

# Step 2 Total RNA Clean-up and RNA QC

**Kits and Reagents:**

RNasey Mini Kit(Qiagen p/n 74104):

Buffer RPE

Buffer RLT

RNeasy Mini spin column

NanoDrop ND-1000

Baseline-ZERO DNase(EPICENTRE, Cat. Nos. DB0711K)

**Things to do before starting:**

1. Buffer RPE is supplied as a concentrate. Before using for the first time, add 4 volumes of ethanol (96–100%) as indicated on the bottle to obtain a working solution.

**Procedure:**

1. Mix the following in a microcentrifuge tube:

| Redissolved RNA | ≤85ul |
| --- | --- |
| 10×Reaction buffer | 10ul |
| Baseline-ZERO DNase | 5ul |
| RNase-free water | X ul |
| **Total volume** | **100ul** |

1. Incubate at 37°C for 30 minutes.
2. Add 350 μl Buffer RLT, and mix well.
3. Add 250 μl ethanol (96–100%) to the diluted RNA, and mix well by pipetting. Do not centrifuge. Proceed immediately to step 5.
4. Transfer the sample (700 μl) to an RNeasy Mini spin column placed in a 2 ml collection tube. Close the lid gently, and centrifuge for 15 s at ≥8000 x g (≥10,000 rpm). Discard the flow-through.
5. Add 500 μl Buffer RPE to the RNeasy spin column. Close the lid gently, and centrifuge for 15 s at≥8000 x g (≥10,000 rpm) to wash the spin column membrane. Discard the flow-through.
6. Add 500 μl Buffer RPE to the RNeasy spin column. Close the lid gently, and centrifuge for 2 min at ≥8000 x g (≥10,000 rpm) to wash the spin column membrane.
7. Place the RNeasy spin column in a new 2 ml collection tube, and discard the old collection tube with the flow-through. Close the lid gently, and centrifuge at full speed for 1 min.
8. Place the RNeasy spin column in a new 1.5 ml collection tube. Add appropriate RNase-free water (please see report ”RNA-QC”) directly to the spin column membrane. Close the lid gently, and centrifuge for 1 min at ≥8000 x g (≥10,000 rpm) to elute the RNA.
9. RNA quantification and quality control(Passed-please see report ”RNA-QC”).

# Step 3 Prepare labeling reaction

**Kit:**

Quick Amp Labeling Kit, One-Color (Agilent p/n 5190-0442)

**Procedure:**

1. Add 1ug of total RNA to a 1.5-mL microcentrifuge tube.
2. Add 1.2 μL of T7 Promoter Primer.
3. Use nuclease-free water to bring the total reaction volume to 11.5 μL.
4. Denature the primer and the template by incubating the reaction at 65°C in a circulating water bath for 10 minutes.
5. Place the reactions on ice and incubate for 5 minutes.
6. Immediately prior to use, gently mix the components listed in the following table for the cDNA Master Mix by adding in the order indicated, and put on ice.

|  | Volume(ul) per reaction |
| --- | --- |
| 5×First Strand Buffer | 4 |
| 0.1M DTT | 2 |
| 10mM dNTP mix | 1 |
| MMLV-RT | 1 |
| RNaseOut | 0.5 |
| **Total volume** | **8.5** |

1. Briefly spin each sample tube in a microcentrifuge to drive down the contents from the tube walls and the lid. Return the tubes to ice.
2. Add 8.5 μL of cDNA Master Mix to each sample tube and mix by pipetting up and down.
3. Incubate samples at 40°C in a circulating water bath for 2 hours.
4. Move samples to a 65°C circulating water bath and incubate for 15 minutes.
5. Move samples to ice. Incubate for 5 minutes.
6. Spin samples briefly in a microcentrifuge to drive down tube contents from the tube walls and lid.
7. Immediately prior to use, gently mix the components listed in the following table in the order indicated for the Transcription Master Mix by pipetting at room temperature.

|  | Volume(ul) per reaction |
| --- | --- |
| Nuclease-free water | 15.3 |
| 4X Transcription Buffer | 20 |
| 0.1 M DTT | 6 |
| NTP mix | 8 |
| 50% PEG | 6.4 |
| RNaseOUT | 0.5 |
| Inorganic pyrophosphatase | 0.6 |
| T7 RNA Polymerase | 0.8 |
| Cyanine-3-CTP | 2.4 |
| **Total volume** | **60** |

1. Add 60 μL of Transcription Master Mix to each sample tube. Gently mix by pipetting.
2. Incubate samples in a circulating water bath at 40°C for 2 hours.

# Step 4 Purify the labeled/amplified RNA and labeled cRNA QC

**Kit and instrument:**

RNeasy Mini Kit (Qiagen p/n 74104)

NanoDrop ND-1000

**Procedure:**

1. Add 20 μL of nuclease-free water to your cRNA sample, for a total volume of 100 μL.
2. Add 350 μL of Buffer RLT and mix well by pipetting.
3. Add 250 μL of ethanol (100% purity) and mix thoroughly by pipetting. Do not centrifuge.
4. Transfer the 700 μL of the cRNA sample to an RNeasy mini column in a 2 mL collection tube. Centrifuge the sample at 4°C for 30 seconds at 13,000 rpm. Discard the flow-through and collection tube.
5. Transfer the RNeasy column to a new collection tube and add 500 μL of buffer RPE (containing ethanol) to the column. Centrifuge the sample at 4°C for 30 seconds at 13,000 rpm. Discard the flow-through. Re-use the collection tube.
6. Add another 500 μL of buffer RPE to the column. Centrifuge the sample at 4°C for 60 seconds at 13,000 rpm. Discard the flow-through and the collection tube.
7. Elute the cleaned cRNA sample by transferring the RNeasy column to a new 1.5 mL collection tube. Add appropriate RNase-free water (Passed-please see report ”Labeling Efficiency-QC”) directly onto the RNeasy filter membrane. Wait 60 seconds, then centrifuge at 4°C for 30 seconds at 13,000 rpm.
8. Maintain the cRNA sample-containing flow-through on ice. Discard the RNeasy column.
9. Take 1.5 μl of each sample to determine the yield and specific activity by using the NanoDrop ND-1000( Passed-please see report ”Labeling Efficiency-QC”).

- From the main menu, choose MicroArray Measurement. Go to the Sample Type pull-down menu and select DNA-50.
- Blank the instrument with 1.5 μL of 1x labeling solution.
- Use 1.5 μL of purified labeled genomic DNA for quantitation. Measure the absorbance at A260nm (DNA), A550nm (cyanine 3).
- The specific activity (pmol dyes per μg genomic DNA) of the labeled genomic DNA can be obtained by the following calculation:

(Concentration of Cy3)

Specific Activity =——————————————— = pmol Cy3 per μg cRNA

(Concentration of cRNA) * 1000

* If the yield is <1.65 μg and the specific activity is <9.0 pmol Cy3 per μg cRNA do not proceed to the hybridization step. Repeat cRNA preparation.

# Step 5 Hybridization

**Kit and Instruments:**

Agilent Gene Expression Hybridization Kit (Agilent p/n 5188-5242):

10X Blocking Agent

25X Fragmentation Buffer

2x GEx Hybridization Buffer HI-RPM

Hybridization Chamber, stainless (Agilent p/n G2534A)

Hybridization Chamber gasket slides (Agilent p/n G2534-60003)

Hybridization oven (Agilent p/n G2545A)

Hybridization oven rotator for Agilent Microarray Hybridization Chambers (Agilent p/n G2530-60029)

**Procedure:**

1. Add 500 μL of nuclease-free water to the vial containing lyophilized 10X Blocking Agent. Mix by gently vortexing.
2. Equilibrate water bath to 60°C.
3. For each microarray, add each of the components as indicated in the tables as below to a 1.5 mL nuclease-free microfuge tube:

| For 1*244K array | Amount |
| --- | --- |
| cyanine 3-labeled, linearly amplified cRNA | 1.5ug |
| 10X Blocking Agent | 50ul |
| Nuclease-free water | ×ul |
| 25X Fragmentation Buffer | 10ul |
| **Total volume** | **250ul** |

| For 2*105K array | Amount |
| --- | --- |
| cyanine 3-labeled, linearly amplified cRNA | 1. 5ug |
| 10X Blocking Agent | 25ul |
| Nuclease-free water | ×ul |
| 25X Fragmentation Buffer | 5ul |
| **Total volume** | **125ul** |

| For 4*44K array | Amount |
| --- | --- |
| cyanine 3-labeled, linearly amplified cRNA | 1.65ug |
| 10X Blocking Agent | 11ul |
| Nuclease-free water | ×ul |
| 25X Fragmentation Buffer | 2.2ul |
| **Total volume** | **55ul** |

| For 8*15K array | Amount |
| --- | --- |
| cyanine 3-labeled, linearly amplified cRNA | 0.6ug |
| 10X Blocking Agent | 5ul |
| Nuclease-free water | ×ul |
| 25X Fragmentation Buffer | 1ul |
| **Total volume** | **25ul** |

1. Mix well but gently on a vortex mixer.
2. Incubate at 60°C for exactly 30 minutes to fragment RNA.
3. Add 2x GEx Hybridization Buffer HI-RPM to the array to stop the fragmentation reaction.

| **Volumes per hybridization** | | | | |
| --- | --- | --- | --- | --- |
| Components | 1*244K | 2*105K | 4*44K | 8*15K |
| cRNA from Fragmentation Mix | 250ul | 125ul | 55ul | 25ul |
| 2* GEx Hybridization Buffer HI-RPM | 250ul | 125ul | 55ul | 25ul |

1. Mix well by careful pipetting. Take care to avoid introducing bubbles. Do not mix on a vortex mixer; mixing on a vortex mixer introduces bubbles.
2. Spin for 1 minute at room temperature at 13,000 rpm in a microcentrifuge to drive the sample off the walls and lid and to aid in bubble reduction.
3. Place sample on ice and load onto the array as soon as possible.
4. Load a clean gasket slide into the Agilent SureHyb chamber base with the label facing up and aligned with the rectangular section of the chamber base. Ensure that the gasket slide is flush with the chamber base and is not ajar.
5. Slowly dispense the volume of hybridization sample onto the gasket well in a “drag and dispense” manner.

| **Volumes per hybridization** | | | | |
| --- | --- | --- | --- | --- |
| Components | 1*244K | 2*105K | 4*44K | 8*15K |
| Volume Prepared | 500ul | 250ul | 110ul | 50ul |
| Hybridization Sample Volume | 490ul | 245ul | 100ul | 40ul |

1. Slowly place an array “active side” down onto the SureHyb gasket slide, so that the “Agilent”-labeled barcode is facing down and the numeric barcode is facing up. Verify that the sandwich-pair is properly aligned.
2. Place the SureHyb chamber cover onto the sandwiched slides and slide the clamp assembly onto both pieces.
3. Hand-tighten the clamp onto the chamber.
4. Vertically rotate the assembled chamber to wet the gasket and assess the mobility of the bubbles.
5. Place assembled slide chamber in rotisserie in a hybridization oven set to 65°C. Set your hybridization rotator to rotate at 10 rpm.
6. Hybridize at 65°C for 17 hours.

# Step 6 Microarray Wash

**Kit and Instruments:**

Gene Expression Wash Buffer 1 (Agilent p/n 5188-5325)

Gene Expression Wash Buffer 2 (Agilent p/n 5188-5326)

Magnetic stir bar (Corning p/n 401435)

Magnetic stir plate (Corning p/n 6795-410)

Slide-staining dish, with slide rack (Thermo Shandon p/n 121)

**Procedure:**

1. Prewarm enough volume of Gene Expression Wash Buffer 2 to 37°C.
2. Add the slide rack and stir bar to the staining dish.
3. Transfer the staining dish with the slide rack and stir bar to a magnetic stir plate.
4. Fill the staining dish with 100% acetonitrile.
5. Turn on the magnetic stir plate and adjust the speed to a setting of 4 (medium speed).
6. Wash for 5 minutes.
7. Discard the acetonitrile as is appropriate for your site.
8. Repeat step 2 to step 7.
9. Air dry the staining dish in the vented fume hood.
10. Wash all dishes, racks, and stir bars with Milli-Q water.
11. Completely fill slide-staining dish #1 with Gene Expression Wash Buffer 1 at room temperature.
12. Place a slide rack into slide-staining dish #2. Add a magnetic stir bar. Fill slide-staining dish #2 with enough Gene Expression Wash Buffer 1 at room temperature to cover the slide rack. Place this dish on a magnetic stir plate.
13. Place the empty dish #3 on the stir plate and add a magnetic stir bar. Do not add the prewarmed (37°C) Gene Expression Wash Buffer 2 until the first wash step has begun.
14. Remove one hybridization chamber from incubator and record time. Record whether bubbles formed during hybridization and if all bubbles are rotating freely.
15. Prepare the hybridization chamber disassembly.
16. Place the hybridization chamber assembly on a flat surface and loosen the thumbscrew, turning counterclockwise.
17. Slide off the clamp assembly and remove the chamber cover.
18. With gloved fingers, remove the array-gasket sandwich from the chamber base by grabbing the slides from their ends. Keep the microarray slide numeric barcode facing up as you quickly transfer the sandwich to slide-staining dish #1.
19. Without letting go of the slides, submerge the array-gasket sandwich into slide-staining dish #1 containing Gene Expression Wash Buffer 1.
20. With the sandwich completely submerged in Gene Expression Wash Buffer 1, pry the sandwich open from the barcode end only:
21. Slip one of the blunt ends of the forceps between the slides.
22. Gently turn the forceps upwards or downwards to separate the slides.
23. Let the gasket slide drop to the bottom of the staining dish.
24. Remove the microarray slide and place into slide rack in the slide-staining dish #2 containing Gene Expression Wash Buffer 1 at room temperature.
25. When all slides in the group are placed into the slide rack in slide-staining dish #2, stir using setting 4 for 1 minute.
26. During this wash step, remove Gene Expression Wash Buffer 2 from the 37°C water bath and pour into the slide-staining dish #3.
27. Transfer slide rack to slide-staining dish #3 containing Gene Expression Wash Buffer 2 at elevated temperature. Stir using setting 4 for 1 minute.
28. Slowly remove the slide rack minimizing droplets on the slides. It should take 5 to 10 seconds to remove the slide rack.
29. Scan slides immediately to minimize the impact of environmental oxidants on signal intensities.

# Step 7 Scanning

**Instrument:**

Agilent Microarray Scanner (Agilent p/n G2565BA)

**Procedure:**

1. Assemble the slides into an slide holder.
2. Place assembled slide holders into scanner carousel.
3. Verify scan settings for one-color scans.

|  | Parameters |
| --- | --- |
| Scan region | Scan Area (61 x 21.6 mm) |
| Scan resolution (μm) | 5 |
| 5μm scanning mode | Single Pass |
| eXtended Dynamic range | (selected) |
| Dye channel | Green |
| Green PMT | XDR Hi 100%  XDR Lo 10% |

1. Click **Scan Slot m-n** on the Scan Control main window where the letter **m** represents the Start slot where the first slide is located and the letter **n** represents the End slot where the last slide is located.

# Step 8 Extract data using Agilent Feature Extraction Software

**Software:**

Agilent Feature Extraction

**Procedure:**

1. Open the Agilent Feature Extraction (FE) software.
2. Add the images (.tif) to be extracted to the FE Project.
3. Set FE Project Properties.
4. Check the Extraction Set Configuration.
5. Save the FE Project (.fep) by selecting **File > Save As** and browse for desired location.
6. Select **Project > Start Extracting** and export data to txt..
